# Renal Endometriosis Mimics Renal Cell Carcinoma in a Hypoplastic Kidney: A Case Report

**DOI:** 10.7759/cureus.55280

**Published:** 2024-02-29

**Authors:** Panagiotis Katsikatsos, Konstantinos Douroumis, Dimitrios Goutas, Harikleia Gakiopoulou, Periklis Anastasiou, Ioannis Anastasiou

**Affiliations:** 1 Urology, Laiko General Hospital, Athens, GRC; 2 Pathology, National and Kapodistrian University of Athens School of Medicine, Athens, GRC; 3 Medicine, University Hospital of Ioannina, University of Ioannina, Ioannina, GRC

**Keywords:** case report, renal endometriosis, renal hypoplasia, renal tumour, endometriosis

## Abstract

*Renal endometriosis* is a rare disorder of cases of urinary tract endometriosis. A 42-year-old woman presented at our outpatient department with an incidental painless mass on her left hypoplastic kidney revealed on an abdominal ultrasound. Abdominal and pelvic examinations revealed no abnormal findings. A computed tomography (CT) scan revealed an anterolateral slightly enhanced left renal mass that measured 1.2 cm in diameter. Furthermore, CT did not reveal any evidence of abdominal or thoracic metastasis. There are a few case reports in the literature of tumors in specimens from patients who underwent nephrectomy for hypoplastic kidneys, but discriminating between benign and malignant masses is difficult unless a nephrectomy is performed. Given the radiological findings and the impaired function of the hypoplastic kidney, laparoscopic radical nephrectomy was recommended. The procedure was performed under general anesthesia without intraoperative or postoperative complications. Microscopic examination revealed several findings consistent with a diagnosis of renal endometriosis. The patient had no symptoms at her last follow-up visit. This case highlights that renal endometriosis can mimic renal cell carcinoma and awareness of this entity should be raised, as it can be asymptomatic, especially when located in a hypoplastic kidney.

## Introduction

Endometriosis is a common gynecologic condition characterized by the implantation of endometrial tissue in extrauterine sites. It is broadly divided into endopelvic and extrapelvic diseases [1]. The incidence of endometriosis varies from approximately 6% to 10% in women of reproductive age, and it commonly manifests as chronic pelvic pain [2]. Extrapelvic sites of endometriosis include the abdominal wall, thorax, gastrointestinal tract, and urinary tract. Endometriosis in the urinary tract is rare and accounts for 1-2% of all cases. The bladder and ureter are the most commonly affected organs, with a prevalence of 85% and 15%, respectively, while the kidney and urethra account for less than 1% [3]. The most common presentations of renal endometriosis are flank pain and gross haematuria, although many cases present with vague, nonspecific symptoms. Hypoplastic kidneys result from multiple contributing factors, such as congenital hypoplasia, chronic urinary tract infections, renovascular ischemia, urological interventions, and surgeries [4]. There are a few case reports in the literature of tumors in specimens from patients who underwent nephrectomy for hypoplastic kidneys. The incidence rate is low, varies among studies, and is associated with patient characteristics [5-7]. We report the case of a 42-year-old woman who underwent laparoscopic left radical nephrectomy for renal endometriosis that was preoperatively misinterpreted as a malignant renal tumor. This report aims to share our experience and raise awareness that pelvic pain in women of childbearing age should be approached with a high level of suspicion to avoid misdiagnosis of renal endometriosis.

## Case presentation

A 42-year-old woman presented to our outpatient clinic for a left kidney mass detected on an abdominal follow-up ultrasound for the atrophic kidney. Her medical history included hypertension and depression, for which she was on medication. Her surgical history was notable for thyroidectomy for papillary thyroid cancer and cervical lymph node dissection for a metastatic lymph node five years ago. The gynecological history was uneventful, with a normal menstruation cycle and no symptoms of dysmenorrhea or dyspareunia. Her family history was remarkable for hypertension and diabetes mellitus from the paternal side and unremarkable on the maternal side. The patient’s medical history included an incidental diagnosis of left kidney hypoplasia 17 years ago on an abdominal ultrasound scan.

A CT scan following the incidental renal hypoplasia finding did not reveal any pathological findings. A dimercaptosuccinic acid (DMSA) scan showed left kidney involvement in renal function of 8%. Patient follow-up included a yearly ultrasound scan.

In the 2022 follow-up ultrasound, a small left renal mass with a diameter of 1 cm was observed. On presentation, the patient did not complain of haematuria, fever, or flank pain. Abdominal and pelvic examinations (transvaginal ultrasound) revealed no abnormal findings from female reproductive organs. The estimated glomerular filtration rate was 69 mL/min/1.73 m^2^. Chest and abdominal CT with an IV contrast agent was ordered.

**Figure 1:**
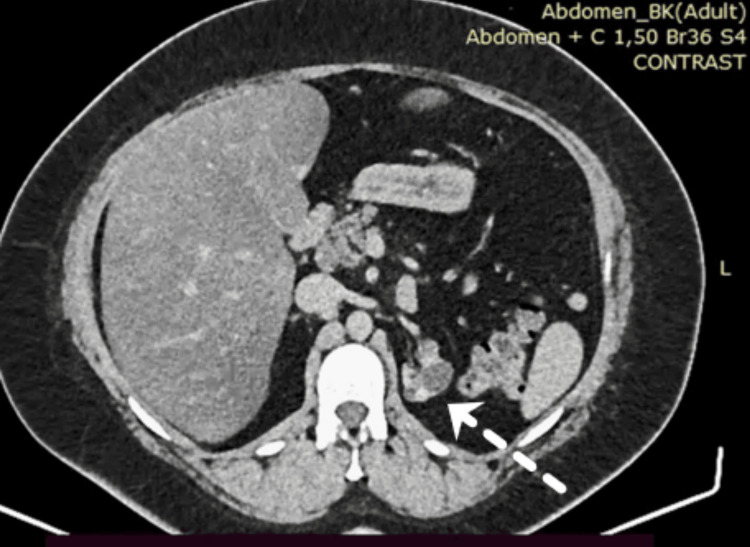
Computed tomography axial view shows a slightly enhanced mass of 1.2 × 1.1 × 0.9 cm on the left hypoplastic kidney, positioned in the renal midline (arrow).

**Figure 2:**
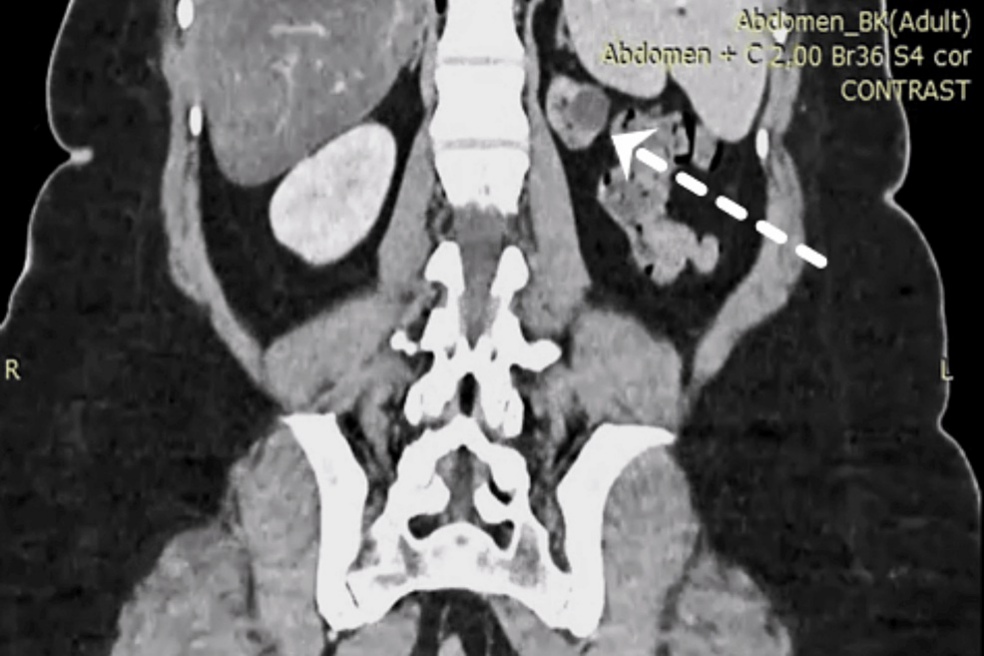
Computed tomography coronal view showing the mass on the left hypoplastic kidney (arrow)

The CT scan showed an anterolateral slightly enhanced mass on the left hypoplastic kidney measuring ~1.2×1.1×0.9 cm (Figures 1-2). The mass was positioned in the renal midline. The central region was measured at 23 Hounsfield units (HU) and was enhanced to 94 HU in the arterial phase. The lesion was categorized as Bosniak IV, according to the Bosniak classification system of renal cystic masses [8]. Furthermore, CT did not reveal any pathological findings from the ovaries and uterus or evidence of abdominal and thoracic metastasis.

A review of the diagnostic findings was presented at a multidisciplinary tumor board meeting. Treatment options included renal biopsy and partial and radical nephrectomy. Given the radiological findings and impaired function of the hypoplastic kidney, laparoscopic radical nephrectomy was recommended.

A laparoscopic left radical nephrectomy was performed under general anesthesia. The patient was placed in a modified flank position, and standard left-sided laparoscopic kidney port placement was performed [9]. The nephrectomy was performed without major intraoperative complications, and the kidney was excised along with Gerota’s fascia. The operation time was 128 minutes. The total blood loss was 133 cc. The specimen was sent for pathologic analysis without being incised intraoperatively. Furthermore, there were no signs of endometriosis in the abdominal cavity during surgery.

**Figure 3:**
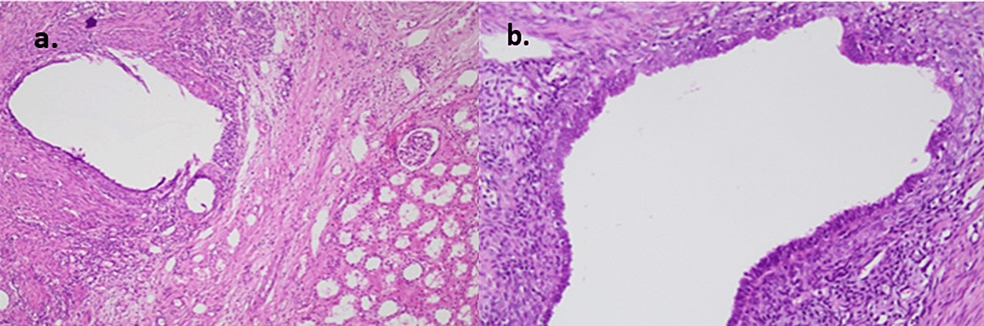
a) Benign endometrial-type glands and stroma adjacent to the renal cortex (H/E x100). b) The benign endometrial type gland is lined by a single layer of columnar cells and endometrial stroma with a fine capillary network (H/E x200)

**Figure 4:**
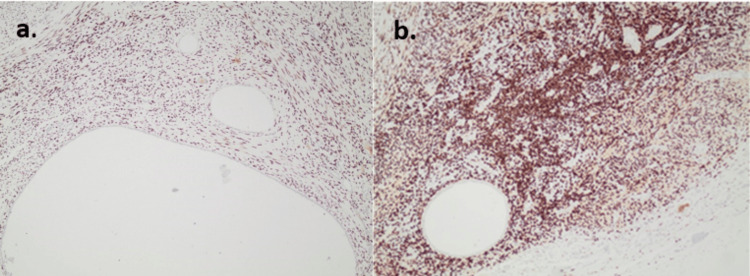
a) Oestrogen receptor (ER) and b) progesterone Receptor (PR) show strong nuclear staining in endometrial stroma and patchy positive staining in benign endometrial glands (x100).

**Figure 5:**
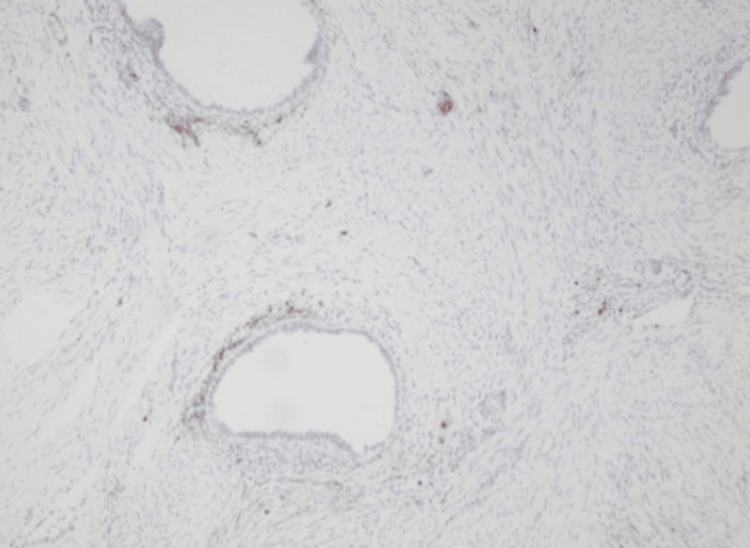
Focal cytoplasmic CD10 staining in endometrial stromal cells (x100)

The postoperative course was uneventful. On postoperative day one, the Foley catheter and redon drainage tube were removed. The patient was discharged on postoperative day two. Microscopic examination of the specimen revealed multiple nodular foci of endometrial glandular and cystic formations lined by a single cell layer of columnar cells and surrounded by endometrial stroma (Figure 3). Immunohistochemical evaluation revealed diffuse and strong nuclear positivity for hormonal receptors (ER and PR) in the benign endometrial glandular epithelium and patchy positive staining in the endometrial stroma (Figure 4), while CD10 staining revealed focal cytoplasmic staining in endometrial stromal cells (Figure 5). These morphologic and immunophenotypic findings were consistent with a diagnosis of renal endometriosis. According to the findings, the patient was subjected to a follow-up protocol, which included a physical examination and an abdominal ultrasound every six months for the first year. At her last visit, the patient remained free of symptoms.

## Discussion

The pathogenesis of extrapelvic endometriosis remains controversial, and several hypotheses, including the ectopic transplantation theory, metaplasia of the coelomic epithelium, autoimmunity, blood lymphatic embolism, and the embryonic theory, have been proposed [10]. The ectopic transplantation theory, as proposed by Sampson in 1927, is generally accepted as the main cause of endometriosis [11], but in some cases such as Mayer-Rokitansky-Kuster-Hauser syndrome, ovarian endometrioma, and peritoneal endometriosis, the coelomic metaplasia theory may be a suitable explanation [10-12]. In the coelomic metaplasia theory, the coelomic epithelium undergoes metaplasia and forms endometrial stroma and glands [13]. The case reported here is difficult to explain by any specific theory.

The presence of genitourinary symptomatology depends on the extent, depth, and location of the ectopic endometrium. Symptoms include the typical triad of renal cell carcinoma (RCC) symptoms, lumbar pain, gross haematuria, and palpable lumbodorsal mass; thus, misdiagnosis can occur in many cases. One specific characteristic of renal endometriosis is repeated periodic pain and haematuria in parallel with the patient’s menstrual cycles [14]. In this case, the patient did not present with any symptoms because the mass was small and confined to the renal cortex with no involvement of the calyces.

The diagnosis of endometriosis is often complicated by the lack of characteristic features on CT and magnetic resonance imaging (MRI), as it shares many similarities with cystic malignancies [13]. According to a literature review by Yang et al. [14] published in 2021, in 16 case reports of renal endometriosis, the preoperative diagnosis ranged from pyelonephritis, hematoma, and renal cyst to renal mass. Clinicians were unable to make a diagnosis in any of these cases without histopathology, revealing the difficulty in identifying this rare entity purely with clinical and imaging criteria [13-30]. In our case, CT failed to distinguish the lesion and showed slight enhancement in the central parts of the lesion. Renal endometriosis should be suspected in young women of reproductive age with incidental masses who present with symptoms that change during the menstrual cycle. In patients with a high clinical suspicion of renal endometriosis, a biopsy via fine-needle aspiration can be performed to alter treatment decisions, with the potential risk of tumor seeding via needle tracks [31].

Close active surveillance can be pursued in patients without symptoms diagnosed via biopsy [15]. For symptomatic endometriosis, medical or surgical treatment is needed to alleviate symptoms. Medical treatment seems to be the best option for patients of reproductive age and reduces pain. The agents that can be used are oral contraceptives and GnRH agonists [20,23,26]. Definitive treatments for renal endometriosis include ablation or partial nephrectomy to alleviate symptoms [16]. In this case, the patient was asymptomatic, and thus, renal endometriosis was not suspected. Due to the high suspicion of renal tumor on the CT scan and the hypoplastic left kidney with minimal participation in renal function, laparoscopic radical nephrectomy was proposed. Discriminating between a benign mass and a malignancy is difficult unless a nephrectomy is performed. A definitive diagnosis was made after the histopathological examination, as in all the abovementioned cases.

After surgical treatment, a follow-up protocol should be established. According to several cases of urinary tract endometriosis [32,33], long-term surveillance with physical examination and abdominal ultrasound should be pursued to evaluate symptom recurrence or anatomic relapse. In our case, we performed a physical examination and abdominal ultrasound every six months for the first year and subsequently every 12 months for the next two years.

Renal endometriosis is a challenging diagnosis that should be suspected in women of childbearing age who exhibit symptoms that change with the menstrual cycle. Radiologic findings are not definitive in the differential diagnosis, and a biopsy should be performed if there is a high suspicion. Especially in cases of hematuria, a suspicion of urothelial carcinoma or a coexistence of RCC with urothelial carcinoma should be suspected [34]. Nephron-sparing techniques, such as ablation and partial nephrectomy, should be performed in these patients, as most of these patients are young, and renal endometriosis is a benign disease.

## Conclusions

We report the case of a woman who presented with a cystic mass in her left hypoplastic kidney that was determined to be renal endometriosis. As imaging fails to diagnose renal endometriosis effectively, the final diagnosis requires histological confirmation. This case shows that renal endometriosis can mimic renal cell carcinoma and awareness of this entity should be raised, as it can be asymptomatic, especially when located on a hypoplastic kidney.
